# Assessment of Cytotoxic and Apoptotic Effects of *Salvia syriaca *L. in Colorectal Adenocarcinoma Cell Line (Caco-2)

**DOI:** 10.22037/ijpr.2020.114254.14759

**Published:** 2021

**Authors:** Şevki Arslan, Kübra Kocabıyık, Doğukan Mutlu, Gürkan Semiz

**Affiliations:** *Department of Biology, Faculty of Arts and Sciences, Pamukkale University, Denizli, Turkey.*

**Keywords:** Salvia syriaca L, Cytotoxic and apoptotic activity, GC-MS, Essential oil, Caco-2

## Abstract

This work is aimed to elucidate cytotoxic and apoptotic effects of *Salvia syriaca *essential oil and its chemical composition by GC-MS. The human colon cancer cells (Caco-2) were treated with different essential oil concentrations for 24 h. Crystal violet test was used to determine cell viability at 630 nm by using an ELISA reader. Apoptotic processes were measured by Annexin V-FITC Apoptosis Assay Kit. Germacrene D (21.77%), trans-β-ocimene (14.66%), β-pinene (9.07%), α-cadinol (8.19%) and α-pinene (6.50%) were the main components of oil determined by GC-MS. Moreover, we observed that the cytotoxic effect was increased with an increasing dose of essential oil. The EC50 value was calculated as 63.5 µg/mL. An increase in the percentage of apoptotic cells was observed after treatment of Caco-2 cells with *S. syriaca* essential oil revealed by image-based cytometry. A nearly 6-fold increase was found in annexin-positive cells after treatment. In terms of mRNA levels, RT-PCR analysis indicated that, although Bax and Caspase-3 were increased, Bcl-2 was decreased after oil treatment. According to our results, *S. syriaca* essential oil has promising phytochemicals that might be useful in cancer treatment due to their relatively cytotoxic and apoptotic activities in Caco-2 cells.

## Introduction

It is well established that most of the plants have been used widely as complementary and alternative therapy. In this regard, Turkey is a very rich country in terms of its flora, including around 12.000 plants species (1). The majority of the Turkish people use most of these plants for different purposes. *Salvia* species are one of the best-known plants used for medical purposes in Turkey as well as in the world. *Salvia* is considered one of the important genus in Lamiaceae with more than 1000 species (2). Most of the *Salvia* species are widely consumed in traditional medicine for the treatment of cold, stomach aches or sore throat, digestive problems and many other problems in Turkey (3-6). They have many different biological activities, including anti-tumoral, anti-carcinogenic, antioxidant, anti-diabetic, anti-inflammatory, anti-rheumatic, antimicrobial and hepatoprotective activities that are due to their high content of secondary metabolites, including flavonoids and terpenoids (7-16). In addition to their biological activities, some *Salvia* species are used in cosmetic and food industries (17). Therefore, *Salvia* species have paid attention due to their economic importance and their wide diverse biological activities. Among the *Salvia* species, *Salvia officinalis* L. (sage) is one of the most widely used and studied species (13, 14). In Turkey, there are 100 *Salvia* species, and 57 of them are endemic (18). That is, Anatolia shows remarkable richness for genus *Salvia* (19). One of the most common species is *Salvia syriaca *L. which is 30-60 (-80) cm, a rhizomatous and perennial herb in Turkey. Its stem is yellowish-green, erect and branched, eglandular-pubescent below and denser above (and rarely glandular). Leaves simple, (5) 6-13 (16) × (3) 4-8 (10) cm, ovate, chordate, rugose; petiole 3-6 cm. Calyx 5-10 mm, tubular, upper lip straight, tridentate. Corolla 8-12 mm, white, tube straight. The species is mainly spread in Inner Anatolia and North of the Middle East, recognized by the tidy habit, small white corolla and regularly ovate leaves (20, 21). Through its wide distribution, there is no deep information about the chemical composition and cytotoxic activity of *S. syriaca *essential oil. In this respect, the main object of the present study was to determine the cytotoxic and apoptotic effects of *S. syriaca* oil in Caco-2.

## Experimental

*Chemicals *

The purchased chemicals were provided from Sigma-Aldrich Chemical Company (St Louis, Missouri, USA): fetal bovine serum (FBS), Dulbecco’s Modified Eagle’s medium (DMEM), bovine serum albumin (BSA). The Assay Kit (Annexin V-FITC Apoptosis, Biovision, CA, USA) was used to evaluate cell apoptosis. The remaining chemicals and solvents used in this study were purchased at the highest grade of purity. 

*Plant material*

*S. syriaca* was collected from Burdur (Southwest of Turkey) during its flowering period (May-July) in 2018. It was taxonomically identified by Prof. Gürkan Semiz from herbarium specimens of the Biology Department, Pamukkale University. Voucher specimens were deposited in the Chemical Ecology Laboratory under code GSE2343. Small cut aerial parts of the plant were air-dried for one week at R.T. 

*Essential oil preparation*

Air-dried plant materials (100 g) were subjected to hydro-distillation for 3 h using a Clevenger apparatus to obtain an oil. Anhydrous sodium sulfate solution was used to dry the obtained oil, and then the oil was kept in a glass vial at 4 °C until usage.

*GC-MS analysis of the essential oil*

About 15 *μ*L of hexane diluted oil (1:100) was subjected to GC-MS analysis by using Agilent Technologies 7820A model GC system equipped with 5975 inert MSD. The samples were analyzed on a 30 m long HP5-MS capillary column (ID 0.25 mm, film thickness 0.25 mm, Hewlett Packard). The column temperature was programmed at 50 °C for 3 min then raised to 250 °C at a rate of 5 °C/min and kept constant at 250 °C for 5 min. The helium (flow rate 1.2 mL/min) as a carrier gas and SCAN technique was used. Wiley 7 MS and NIST02 libraries were used for the identification of the compounds based on comparing the mass spectral data and known compounds in the literature. The percentages were calculated from GC peaks with the normalization method. 

*Cell culture and cytotoxicity assay*

Caco-2 cells provided from the European Collection of Animal Cell Culture (ECACC, UK) were used in this study. The cells were grown in a DMEM medium with 10% FBS, 1% of *L*-glutamine, 50 *μ*g/mL streptomycin, and 50 IU/mL penicillin in a CO_2_ incubator. 5000 cells/well were sown to 96 well-plate. After 24 h, different concentrations of* S. syriaca* essential oil (1 µg/mL and 100 µg/m l9 were applied to cells. Dimethylsulfoxide (DMSO) was used for the preparation of oil solutions, and its concentration did not exceed 0.5%. For control cells, 0.5% DMSO concentration was also applied. After incubation of another 24 h, medium containing floating cells were taken out, and crystal violet solution was used to stain attached cells. The final color was measured at 630 nm. The absorbance values were used to calculate the EC_50 _concentration of the *S. syriaca* oil. This cytotoxicity experiment was repeated three times to determine the EC_50_ value.

*Apoptosis*

Cells were sown in six-well plates at a concentration of 2 × 10^5 ^cells/wells and exposed to essential oils (EC_50_ doses). H_2_O_2_ was used as a positive control. After 24 h, cells were gathered and stained by using Annexin V-FITC Apoptosis Assay Kit (Biovision, CA, USA) as described in the product manual. For each treatment, 25 μL of cell suspension was loaded into one Arthur™ image-based cytometer slide and analyzed. For each treatment, 20 fields were analyzed, and around 7000 cells were counted. The percentage of apoptotic cells was calculated over the total cell population. This experimental set-up was performed two times, and the apoptotic cell percentage was represented as the mean of independent experimental sets ± SEM.

*RNA isolation and determination of mRNA expression by RT-PCR*

Caco-2 cells were treated with an EC_50_ dose of *S. syriaca* essential oil and incubated for 24 h. Then, cells were collected, and RNA was isolated by using InnuPREP RNA Mini Kit 2.0 (Analytic Jena, Germany) followed the instructions. Agarose gel electrophoresis was used to check the integrity of isolated RNA. The concentration of isolated RNAs was determined by measuring optical density (A260/A280 ratio). OneScript® Plus cDNA Synthesis Kit (ABM, USA) was used for cDNA synthesis. The reaction mixture consisted of 2 μg RNA, 0.5 μM oligo(dT)18 reverse transcription primer, dNTP, RT buffer, RNaseOFF Ribonuclease Inhibitor (20 Units), OneScript® Plus RTase (200 Units) and nuclease-free water. Quantitative real-time PCR assays for Bax, Bcl-2 and Caspase-3 were done by using Applied Biosystems™ StepOnePlus™ Real-Time PCR System (Thermo, USA). RT-PCR reactions were carried out in 10 µL volumes using ABM KiloGreen 2x qPCR MasterMix (ABM, USA). All samples were performed in duplicates. Nuclease-free water was used as a negative control. For the determination of fold changes in mRNA levels, the 2^−ΔΔCt^ method was used as described previously (22). β-*actin*, a reliable housekeeping gene, was used as an internal control.

*Statistical analysis*

The results were given as means ± SD of at least three replicates. Minitab statistical software was used for statistical analyses. For comparisons of the groups, Student’s *t*-test was used. *p *< 0.05 was preferred for statistical significance.

## Results and Discussion

The chemical composition of *S. syriaca* oil was given in Table 1. The obtained oil was pale yellow with a strong smell and a yield of 0.14%. A total of twenty-nine compounds have been identified, corresponding to 99.28% of the total composition of *S. syriaca *essential oil.

 The main compounds were found as: germacrene-D (21.77 %), *trans*-β-ocimene (14.66%), β-pinene (9.70%), α-cadinol (8.19%), α-pinene (6.50%) and γ-cadinene (6.40%). In this study, we first time showed cytotoxic and apoptotic effects of *S. syriaca* essential oil on colon cancer cell line. In current literature, there have been few studies about the chemical composition of *S. syriaca *essential oils. Previously, Turkish *S. syriaca* oils were characterized by germacrene-D (33.83%) and bicyclogermacrene (12.47%) as the most important components (23). Even the major compound, namely germacrene-D, was found in both studies, the composition of the oils was totally different. Furthermore, twenty-two compounds were determined in the oil of *S. syriaca* obtained from Iran and germacrene-B (34.8%), germacrene-D (29.2%), α-ylangene (3.6%) and spathulenol (3.4%) were the main compounds (24). In a study from Jordan, the main compounds were found to be thymol, α-pinene and isobornyl acetate for *S. syriaca* (25). In another study from the West Azerbaijan Province (Iran), it was found that the main compounds of *S. syriaca* were 1,8-cineole (46.45%), camphor (27.58%) and bornyl acetate (4.66%) (26). It is well established that the chemical composition of plant essential oils was changed due to genetic differences, weather/soil conditions, time of harvest, the drying technique, *etc*. Due to these important factors, the chemical composition of *S. syriaca *obtained from this study differed from the other studies. In our study, the most abundant component was identified as germacrene-D (21.77%). The biological function of germacrene-D is not known completely in plants. It was thought that germacrene-D is responsible for the productions of other compounds in different organisms (27, 28) and also in plant-herbivore interactions as a deterrent (29, 30). The essential oils contain *β*-ocimene as a major compound (the second most abundant compound in our study) showed cytotoxic and anti-carcinogenic effects on different cell lines, including Caco-2 (31, 32). 

**Figure 1:**
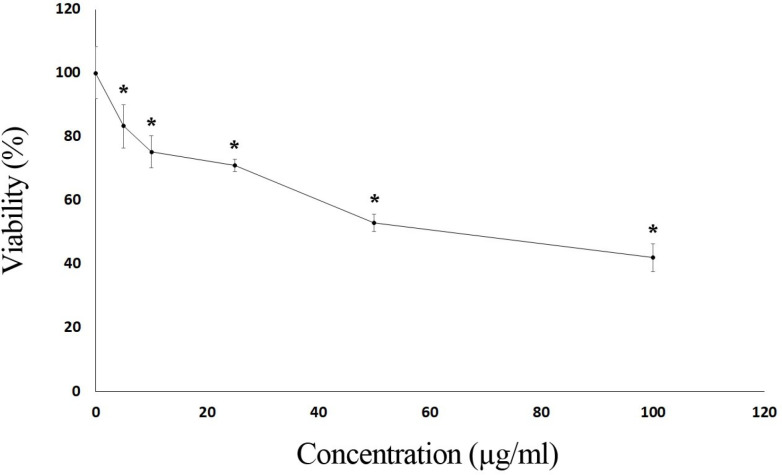
Cytotoxic effects of *S. syriaca* essential oil in CaCo-2 cells. Cells were exposed to different concentrations of essential oil for 24 h. Results are mean ± SD values for three independent experiments (*p < 0.05)

In addition to the determination of chemical content of essential oil, the cytotoxic activity of *S. syriaca* oil was determined in the Caco-2 cell line. Caco-2 cells are important in testing cytotoxic activities of chemicals and experimental pharmacology studies. Moreover, it is well established that *Salvia *species are consumed by preparing tea using their leaves. Due to these reasons, Caco-2 cell lines were selected in this study. As seen in Figure 1, essential oil showed a cytotoxic effect on Caco-2 cells in a dose-dependent manner, and toxicity started at 5 μg/mL.

The EC_50_ value of the essential oil obtained from *S. syriaca* was found to be 63.5 µg/ml. The extracts prepared using different parts of *S. syriaca* and different solvents showed similar cytotoxic effects (33, 34).

**Figure 2:**
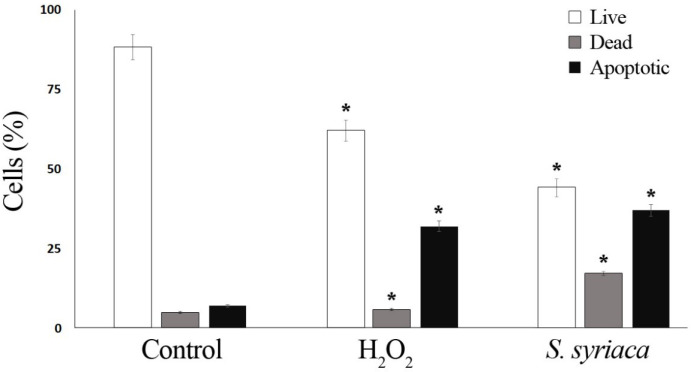
Apoptotic effects of *S. syriaca* essential oil by image-based cytometer. Caco-2 cells were assessed for apoptosis after a 24-hour incubation period with oil. H_2_O_2_ (25 mM) was applied as a positive control (*p < 0.05)

**Figure 3:**
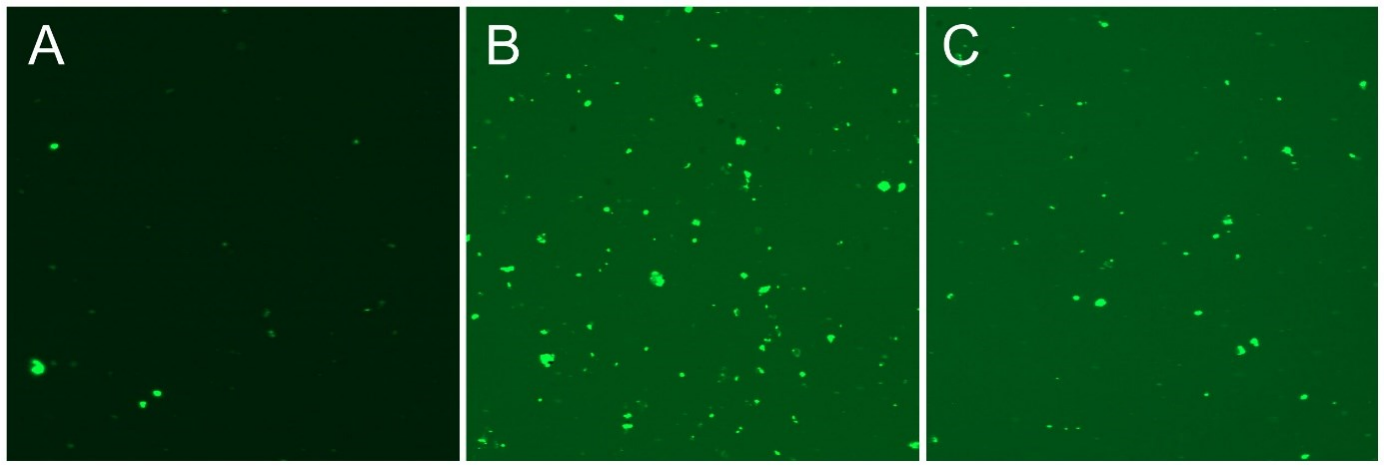
Annexin V staining of Caco2 cancer cells for 24 h with EC_50_ concentration of *S. syriaca* essential oil. (A) Control, (B) H_2_O_2 _treated and (C) *S. syriaca* treated cells

Aqueous crude extract obtained from *S. syriaca* showed a cytotoxic effect, and EC_50_ value was found 94.85 µg/mL (35). Moreover, roots methanol extract of *S. syriaca* showed a cytotoxic effect on Caco-2 cells (34). As seen in these studies, EC_50_ values were different in each study due to their chemical content differences in extract or extraction methods. Similar to our results, essential oils obtained from other *Salvia* species showed cytotoxic activity in different cancer cells (35-37). However, in a comparative study, Firuzi *et al.* (2013) reported different extracts of *S. syriaca* had no or little effect on different human cancer cell lines among the other tested species (38). In order to understand the mechanisms of cytotoxic effect, apoptosis analysis was performed by applying EC_50_ dose to Caco-2 cells in this study. It is well established that one of the important strategies to treat cancer is the induction of apoptosis in tumor cells. Plant extracts or pure compounds obtained from extracts have been shown to induce apoptosis in different cancer cells. Therefore, screening of apoptosis in plant extracts is so important. As given in the method part, apoptosis was determined by Arthur image-based cytometer and the cell proportions were quantified as live, dead and apoptotic cells (Figures 2 and 3). H_2_O_2_, the apoptosis-inducing agent, was chosen as the positive control.

**Figure 4:**
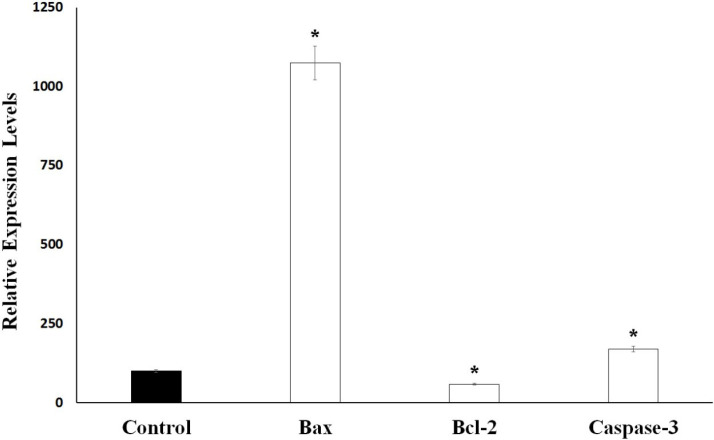
Bax, Bcl-2 and Caspase-3 mRNA levels in control and *S. syriaca* essential oil-treated Caco-2 cells. Individual gene expression levels were normalized by using *β-actin*. The value obtained from control cells was taken to be 100%, and the values obtained from the *S. syriaca *oil-treated cells were expressed as a percentage of control (*p < 0.05)

 As shown in Figure 2, after 24 h of application with essential oil, 37% of the Caco-2 cells were apoptotic, 45% are viable, and 17% are dead. The ratio of apoptotic cell population in *S. syriaca* treatment was approximately 6-fold higher than the control. These results clearly showed that essential oil caused induction of apoptosis and have a cytotoxic effect. Also, in treated cells, a significant decrease was noticed in the percentage of live cells with the comparison to the control ones (Figures 2 and 3). Besides, RT-PCR results showed that *S. syriaca* essential oil increased the pro-apoptotic Bax (10.74-fold) and decreased anti-apoptotic Bcl-2 (2.45-fold) mRNA levels (Figure 4). Oil treatment increased Bax/Bcl-2 ratio as well. The essential oil also caused a 1.7-fold increase in Caspase-3 mRNA level (Figure 4). It is well known that the two main pathways of apoptosis are extrinsic and intrinsic pathways. Each pathway requires different caspase enzymes that cause activation of these pathways by Caspase-3. Based on the results obtained from our study, the intrinsic pathway of apoptosis was involved in *S. syriaca*-induced cell death. 

Similar to our observation, other *Salvia* species also showed apoptotic effects in different cell lines (39-40). 

**Table 1:** The chemical composition of the essential oil obtained from *S. syriaca*

**No**	Compounds*	***RI ***	**(%)**	**No**	**Compounds**	***RI ***	**(%)**
1	tricyclene	919	1.05	16	myrtenal	1189	0.30
2	*α*-pinene	933	6.50	17	*α*-terpineol	1198	0.46
3	camphene	948	1.33	18	*trans*-carveol	1217	0.05
4	*β*-pinene	967	9.70	19	bornyl acetate	1281	0.38
5	myrcene	988	1.18	20	bicycloelemene	1331	0.24
6	3-carene	1014	0.29	21	*α*-cubenene	1357	0.48
7	*α*-terpinene	1017	4.32	22	*α*-copaene	1377	2.23
*8*	*d*-limonene	1033	1.85	23	*β*-elemene	1388	3.27
9	*trans*-*β*-ocimene	1047	14.66	24	*α*-cedrene	1416	1.41
10	*α*-terpinolene	1086	4.28	25	*trans*-*β*-caryophyllene	1423	4.93
11	linalool	1099	1.11	26	germacrene-D	1479	21.77
12	*trans*-pinocarveol	1144	0.28	27	γ-cadinene	1524	6.40
13	camphor	1150	0.13	28	*α*-calacorene	1536	0.40
14	borneol	1165	1.25	29	*α*-cadinol	1650	8.19
15	terpinene-4-ol	1176	0.76		Total		99.28

^*^All the compounds are arranged in order to their elution times on the column. The major compounds are highlighted in bold. *RI*: Retention indices on the HP-5MS column relative to C_8_ to C_24 _*n*-alkanes.

## Conclusion

This is the first record of the cytotoxicity and apoptotic effects of *S. syriaca* in Caco-2 cells. The oil obtained from *S. syriaca* has promising phytochemicals that may be used in cancer treatment. Moreover, an activity-guided fractionation experiment should be done to test our hypothesis. Also, further works are necessary to find the molecule (s) responsible for cytotoxic and apoptotic effects of *S. syriaca*.
